# A Comparison of Virulence of Three Porcine Circovirus Type 2 (PCV2) Genotypes (a, b, and d) in Pigs Singularly Inoculated with PCV2 and Dually Inoculated with PCV2 and Porcine Reproductive and Respiratory Syndrome Virus

**DOI:** 10.3390/pathogens10070891

**Published:** 2021-07-14

**Authors:** Jeongmin Suh, Taehwan Oh, Keehwan Park, Siyeon Yang, Hyejean Cho, Chanhee Chae

**Affiliations:** Department of Veterinary Pathology, College of Veterinary Medicine, Seoul National University, Gwanak-ro 1, Gwanak-gu, Seoul 08826, Korea; tobin1210@snu.ac.kr (J.S.); ohth93@gmail.com (T.O.); kylinhp@snu.ac.kr (K.P.); didtldus12@gmail.com (S.Y.); hcho21@snu.ac.kr (H.C.)

**Keywords:** porcine circovirus-associated disease, porcine circovirus type 2, PCV2 genotypes, porcine reproductive and respiratory syndrome virus

## Abstract

The aim of this study was to compare the virulence of porcine circovirus type 2 (PCV2) genotypes in dually inoculated pigs with both three genotypes (a, b, and d) of PCV2 and porcine reproductive and respiratory syndrome virus-2 (PRRSV-2) versus pigs singularly inoculated with the same three PCV2 genotypes (a, b, and d). Differences in this comparison were found in PCV2 viremia levels, lung and lymphoid lesion severity, and the amount of PCV2 antigen within the lymphoid lesions. Regardless of PCV2 genotypes, pigs that were dually inoculated with PCV2/PRRSV had significantly higher clinical scores, less average daily weight gain, higher levels of PCV2 viremia, and more severe lug and lymphoid lesions compared to pigs singularly inoculated with PCV2. Among the dually infected pig groups, pigs infected with PCV2d/PRRSV-2 had significantly higher levels of PCV2 viremia, more severe lung and lymphoid lesions, and more PCV2-positive cells within lymphoid lesions compared to pigs dually inoculated with PCV2a/PRRSV-2 and PCV2b/PRRSV-2. The results of this study demonstrated significant differences in the virulence among dual inoculation of PCV2a/PRRSV-2, PCV2b/PRRSV-2, and PCV2d/PRRSV-2. A significant difference in the virulence among PCV2a, PCV2b, and PCV2d single-inoculated pig groups was not found with respect to the levels of PCV2 viremia and production of PCV2-associated lymphoid lesions.

## 1. Introduction

Porcine circovirus type 2 (PCV2) and porcine reproductive and respiratory syndrome virus (PRRSV) continues to be the most challenging porcine pathogens today and are the cause of enormous economic losses in the global swine industry. PCV2 is a nonenveloped single-stranded circular DNA virus belonging to the genus *Circovirus* in the family *Circoviridae* [1]. At least eight distinct genotypes (PCV2a to PCV2h) have been identified [2]. PCV2 is the primary etiological agent of several diseases and syndromes collectively referred to as porcine circovirus-associated disease (PCVAD) [3].

PRRSV is an enveloped single-stranded positive-sense RNA virus belonging to the genus *Porarterivirus* in the family *Arteriviridae* [4]. PRRSV was further divided into two species, PRRSV-1 (formerly known as the European genotype 1) and PRRSV-2 (formerly known as the North American genotype 2) [4]. Infection with PRRSV can cause reproductive failure (abortions, weak and stillborn piglets, and infertility) in sows and respiratory disease in weaned and growing pigs [5].

Currently, PCV2a, PCV2b, and PCV2d are the three major genotypes [2]. Among these, PCV2d is the most predominant genotype currently circulating in Asian and North American pig populations, followed by PCV2b and PCV2a [6,7,8]. In the comparison of virulence, the Chinese PCV2d strain caused more serious disease compared to the PCV2a and PCV2b strains while the Korean and North American PCV2d strain exhibited similar virulence to PCV2a and PCV2b strains [9,10,11]. These previous comparative studies were conducted with single infections only [9,10,11]. The results may therefore be limited as although PCV2 is the primary agent, additional pathogens are required to cause PCVAD. Co-infection of pigs with PCV2 and PRRSV is the most commonly combined infection that causes the full spectrum of clinical signs and lesions associated with PCVAD, in particularly, in non-PCV2 vaccinated herds [12,13,14,15,16,17]. This is the reason why it is necessary to conduct the dual infection with PCV2 and PRRSV for the comparison of virulence among three major genotypes. The objective of the present study was to compare the virulence of the three major PCV2 genotypes (2a, 2b, and 2d) in pigs dually infected with PCV2a and PRRSV-2, PCV2b and PRRSV-2, and PCV2d and PRRSV-2, along with pigs singularly infected with PCV2a, PCV2b, and PCV2d.

## 2. Results

### 2.1. Clinical Signs

**Figure 1:**
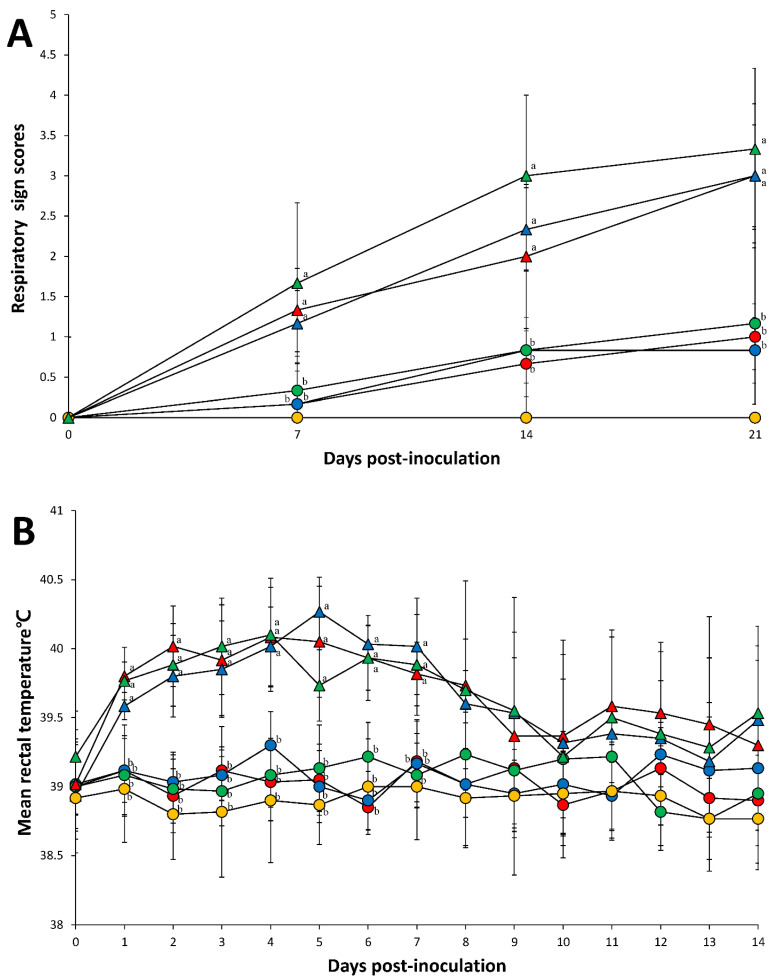
Respiratory sign scores (**A**) and rectal temperature (**B**) of pigs dually inoculated with PCV2a/PRRSV-2 (▲), PCV2b/PRRSV-2 (▲), and PCV2d/PRRSV-2 (▲), and pigs singularly inoculated with PCV2a (●), PCV2b (●), and PCV2d (●), and negative control pigs (●). Variation is expressed as the standard deviation. Different superscripts (a and b) indicate significant (*p* < 0.05) difference among 7 groups.

Mild clinical respiratory sign (tachypnea) was observed in the pigs singularly inoculated with PCV2a, PCV2b, or PCV2d only. Clinical disease was observed in all pigs dually inoculated with either PCV2a/PRRSV-2, PCV2b/PRRSV-2, or PCV2d/PRRSV-2. Regardless of the PCV2 genotype, pigs dually inoculated with PCV2/PRRSV developed severe respiratory distress by 7 dpi (days post-inoculation) that continued through 21 dpi. The respiratory sign scores at 7 to 21 dpi in pigs dually inoculated with PCV2a/PRRSV-2, PCV2b/PRRSV-2, and PCV2d/PRRSV-2 were significantly higher (*p* < 0.05) compared with those of pigs singularly inoculated with PCV2a, PCV2b, or PCV2d only and those of negative control pigs. Tachypnea and pronounced abdominal breathing were first observed at 5 dpi. No clinical respiratory signs were observed in the negative control pigs throughout the entire experiment (Figure 1A).

Mean rectal temperatures were heightened during the period of maximal clinical disease until 7 dpi, after which point the temperatures remained normal. Rectal temperature at 1 to 7 dpi in pigs dually inoculated with PCV2a/PRRSV-2, PCV2b/PRRSV-2, and PCV2d/PRRSV-2 were significantly higher (*p* < 0.05) compared with those of pigs singularly inoculated with PCV2a, PCV2b, or PCV2d only and those of negative control pigs (Figure 1B).

### 2.2. Growth Performance

A statistical difference was not observed at the start of the experiment (42-day-old pigs) in terms of average body weight among the seven groups. Pigs dually inoculated with PCV2a/PRRSV-2, PCV2b/PRRSV-2, and PCV2d/PRRSV-2 had significantly lower average body weights compared to pigs inoculated with either PCV2a, PCV2b or PCV2d alone, as well as negative controls at 63 days old (21 dpi). Pigs dually inoculated with PCV2a/PRRSV-2, PCV2b/PRRSV-2, and PCV2d/PRRSV-2 had a significantly lower (*p* < 0.05) ADWG between 42 and 63 days of age compared to pigs inoculated with either PCV2a, PCV2b, PCV2d, or the negative controls (Table 1).

### 2.3. Enzyme-Linked Immunosorbent Assay

**Figure 2:**
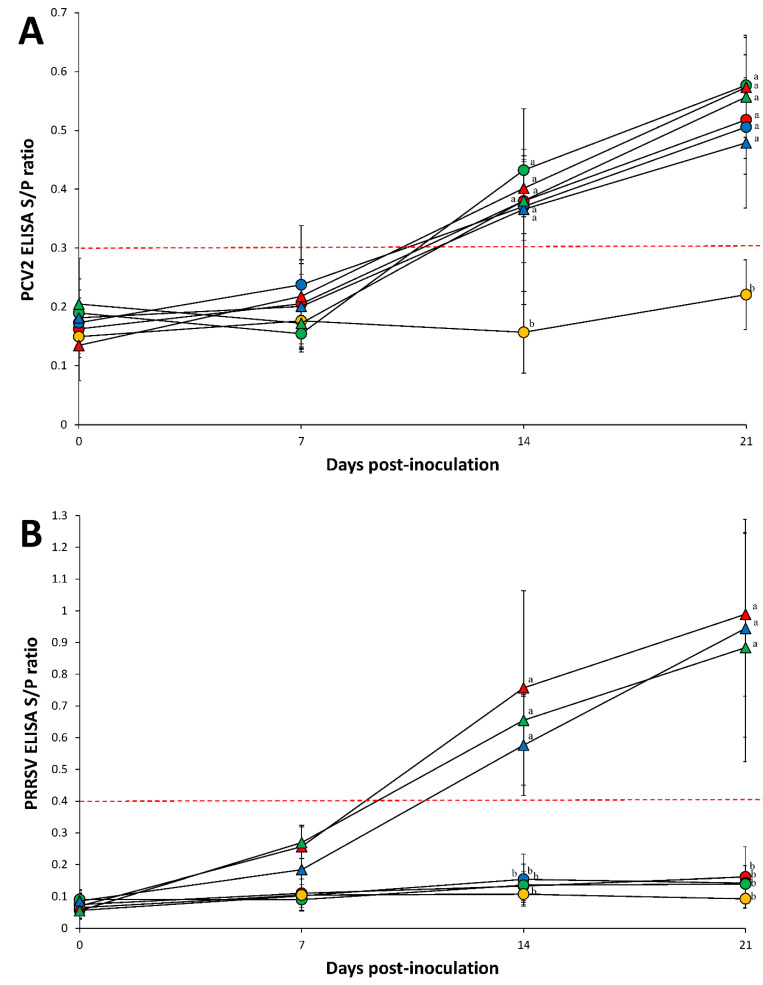
Porcine circovirus type 2 (PCV2)-specific ELISA antibody levels (**A**) and porcine reproductive and respiratory syndrome virus (PRRSV)-specific ELISA antibody levels (**B**) of pigs dually inoculated with PCV2a/PRRSV-2 (▲), PCV2b/PRRSV-2 (▲), and PCV2d/PRRSV-2 (▲), and pigs singularly inoculated with PCV2a (●), PCV2b (●), and PCV2d (●), and negative control pigs (●). Variation is expressed as the standard deviation. Different superscripts (a and b) indicate significant (*p* < 0.05) difference among 7 groups.

There were no significant differences in PCV2 S/P ratio between pigs singularly inoculated with PCV2 and pigs dually inoculated with PCV2 and PRRSV (Figure 2A). PCV2 antibodies were not detected in negative control pigs throughout the entire experiment. PRRSV antibodies were detected in pigs dually inoculated with PCV2 and PRRSV. There were no significant differences in PRRSV S/P ratio between pigs dually inoculated with PCV2 and PRRSV (Figure 2B).

### 2.4. Quantification of PCV2 DNA in the Blood

**Figure 3:**
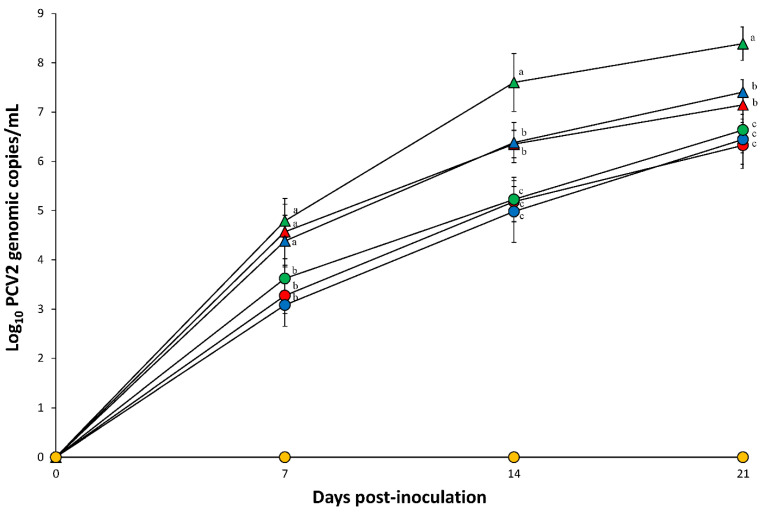
Mean values of the genomic copy number of porcine circovirus type 2 (PCV2) DNA in serum of pigs dually inoculated with PCV2a/PRRSV-2 (▲), PCV2b/PRRSV-2 (▲), and PCV2d/PRRSV-2 (▲), and pigs singularly inoculated with PCV2a (●), PCV2b (●), and PCV2d (●), and negative control pigs (●). Variation is expressed as the standard deviation. Different superscripts (a, b, and c) indicate significant (*p* < 0.05) difference among 7 groups.

Prior to challenge, all serum samples collected from pigs in seven groups tested negative for PCV2a, PCV2b, and PCV2d. After PCV2 challenge, PCV2 genomic copies were detected in the blood at 7, 14, and 21 dpi in all pigs in the dual inoculated (PCV2a/PRRSV-2, PCV2b/PRRSV-2, and PCV2d/PRRSV-2) and single infected (PCV2a, PCV2b, and PCV2d) groups. Pigs dually inoculated with PCV2a/PRRSV-2, PCV2b/PRRSV-2, and PCV2d/PRRSV-2 had a significantly (*p* < 0.05) higher number of PCV2 genomic copies compared to pigs singularly inoculated with either PCV2a, PCV2b, or PCV2d at 7, 14, and 21 dpi. Pigs dually inoculated with PCV2d/PRRSV-2 had a significantly (*p* < 0.05) higher number of PCV2 genomic copies compared to pigs dually inoculated with PCV2a/PRRSV-2 and PCV2b/PRRSV-2 at 14 and 21 dpi (Figure 3). PCV2 genomic copies were not detected in negative control pigs throughout the entire experiment.

### 2.5. Quantification of PRRSV cDNA in the Blood

Prior to challenge, all serum samples collected from pigs in seven groups tested negative for PRRSV. After PRRSV challenge, PRRSV-2 genomic copies were detected in the blood at 7, 14, and 21 dpi in all pigs in the dual inoculated (PCV2a/PRRSV-2, PCV2b/PRRSV, and PCV2d/PRRSV) groups. No significant differences in the number of PRRSV-2 genomic copies were detected in pigs in the PCV2a/PRRSV-2, PCV2b/PRRSV-2, and PCV2d/PRRSV-2 groups. PRRSV-2 genomic copies were not detected in negative control pigs throughout the entire experiment.

### 2.6. Histopathology

**Figure 4:**
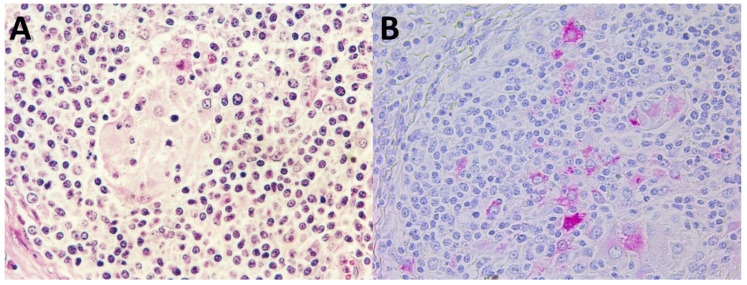
Granulomatous foci characterized by infiltration with reactive histiocytes and multinucleated giant cells in lymph node from pigs dually inoculated with PCV2d/PRRSV-2 (**A**). PCV2 antigens were detected in multinucleated giant cells and histiocytes in lymph node of pigs dually inoculated with PCV2d/PRRSV-2 (**B**).

Minimal histopathologic lesions were seen in piglets singularly inoculated with either PCV2a, PCV2b, or PCV2d. Histopathologic lesions in pigs dually inoculated with PCV2a/PRRSV-2, PCV2b/PRRSV-2, and PCV2d/PRRSV-2 were severe and widespread in the lymph nodes. The primary lesion was widespread disseminated granulomatous inflammation found mainly in lymph nodes and spleens, and occasionally in kidneys. A moderate to severe infiltration of lymphocytes, neutrophils, and eosinophils accompanied macrophages. Lymph nodes were depleted of mature lymphocytes; germinal centers were reduced or absent. Multinucleated giant cells were prominent in pigs dually inoculated with PCV2a/PRRSV-2, PCV2b/PRRSV-2, and PCV2d/PRRSV-2 (Figure 4A). Pigs dually inoculated with PCV2a/PRRSV-2, PCV2b/PRRSV-2, and PCV2d/PRRSV-2 had interstitial pneumonia. Alveolar septa were markedly thickened by infiltrates of macrophages, lymphocytes, and plasma cells. Alveolar septa were diffusely lined by type 2 pneumocytes. Alveolar spaces contained small amounts of necrotic debris. Tissue samples from negative control pigs were histologically normal.

Pigs dually inoculated with PCV2a/PRRSV-2, PCV2b/PRRSV-2, and PCV2d/PRRSV-2 had significantly higher (*p* < 0.05) microscopic lung lesion scores compared to pigs singularly inoculated with PCV2a, PCV2b, and PCV2d alone at 21 dpi. Pigs dually inoculated with PCV2d/PRRSV-2 had significantly higher (*p* < 0.05) microscopic lung lesion scores compared to pigs dually inoculated with PCV2a/PRRSV-2 and PCV2b/PRRSV-2 at 21 dpi (Table 2).

Pigs dually inoculated with PCV2a/PRRSV-2, PCV2b/PRRSV-2, and PCV2d/PRRSV-2 had significantly higher (*p* < 0.05) microscopic lymphoid lesion scores compared to pigs singularly inoculated with PCV2a, PCV2b, and PCV2d alone at 21 dpi. Pigs dually inoculated with PCV2d/PRRSV-2 had significantly higher (*p* < 0.05) microscopic lymphoid lesion scores compared to pigs dually inoculated with PCV2a/PRRSV-2 and PCV2b/PRRSV-2 at 21 dpi (Table 2).

### 2.7. Immunohistochemistry

Regardless of the PCV2 genotype, all pigs infected with PCV2, either alone or in combination with PRRSV reacted positively for PCV2 antigen. In lymph nodes, prominent accumulations of viral antigen were detected chiefly in follicular macrophages in collapsed germinal centers (Figure 4B). Pigs dually inoculated with PCV2a/PRRSV-2, PCV2b/PRRSV-2, and PCV2d/PRRSV-2 had a significantly higher (*p* < 0.05) number of PCV2-positive cells per unit of tissue in their lymph nodes than pigs singularly inoculated with PCV2a, PCV2b, or PCV2d. Pigs dually inoculated with PCV2d/PRRSV-2 had a significantly higher (*p* < 0.05) number of PCV2-positive cells per unit of tissue in their lymph nodes than pigs dually inoculated with PCV2a/PRRSV-2 and PCV2b/PRRSV-2 (Table 2). PCV2 antigens were not detected in any lymph nodes examined in negative control pigs.

### 2.8. Correlation between PCV2 Viremia and Lymphoid Lesions

Positive and significant correlations were found in pigs dually infected with PCV2 and PRRSV groups (PCV2a/PRRSV-2: R = 0.926, *p* < 0.05; PCV2b/PRRSV-2: R = 0.837, *p* < 0.05; PCV2d/PRRSV-2: R = 0.833, *p* < 0.05) between PCV2 viral blood load and the severity of lymphoid lesions.

## 3. Discussion

In the current study, regardless of PCV2 genotypes, pigs dually infected with PCV2/PRRSV developed PCVAD, whereas none of the pigs singularly infected PCV2 alone developed PCVAD. Moreover, significant differences in virulence were found in pigs dually infected with PCV2a/PRRSV-2, PCV2b/PRRSV-2, or PCV2d/PRRSV-2 whereas no significant differences in virulence were found in pigs infected solely with either PCV2a, PCV2b, or PCV2d. The PCV2d strain was more virulent than either the PCV2a or PCV2b strains in a dual infection model, as evident by the increased amount of PCV2 viral blood load and a greater severity in lymphoid lesions. This experimental study does not support major differences in the virulence of PCV2a/PRRSV-2 and PCV2b/PRRSV-2 in a dual infection model. These results agree with previous findings that demonstrated no significant differences in the virulence of PCV2a/PRRSV-2 and PCV2b/PRRSV-2 [18,19]. The single infection model resulted in an inconsistent difference in virulence between the different genotypes. The Chinese PCV2d strain can result in more severe disease compared to either the PCV2a or PCV2b strains [10]. These findings contrast with the single infection model, where three PCV2 genotypes showed similar virulence in the present and previous studies with Korean and North American isolates [9,11].

PRRSV potentiates PCV2 replication, regardless of the PCV2 genotype coinfection. Replication of PCV2 by PRRSV can be influenced by pro-inflammatory cytokines [20]. Different results could have been obtained in a different strain since different PRRSV strains differ significantly in pro-inflammatory responses [21]. PCV2 replication can be enhanced by interferon-γ in vitro [20], which increased in level in pigs infected with PRRSV [22]. In addition, interferon-mediated enhancement of PCV2 replication is decreased by mutation of the PCV2 interferon-stimulated response element (ISRE) in infected pigs [23]. The role of ISRE is further assessed in pigs co-infected with PCV2 and PRRSV. The ISRE mutant of PCV2 reduced viral replication during early stage of co-infection, while the ISRE mutant of PCV2 increased viral replication during late stage of co-infection [24]. Therefore, the enhancement of PCV2 replication by PRRSV is complex and further studies are necessary to elucidate the mechanisms of PRRSV to enhance the PCV2 replication. Nevertheless, the mechanisms underlying the increased virulence of PCV2d strains, compared with that of PCV2a and PCV2b are uncertain in the dual infection model. The increased virulence is tentatively attributed to an extra lysine (K) residue in the capsid protein encoded by ORF2, making it 234 amino acids (aa) long, as compared to the 233 aa capsids of PCV2a and b strains [10]. Moreover, recombination events, especially those involving the ORF2, may influence the antigenicity and virulence of PCV2 [25,26]. Therefore, it may be speculated that PCV2d replicates more efficiently in the pro-inflammatory responses induced by PRRSV infection due to the genetic difference in PCV2d and the other two PCV2 genotypes. Further studies are necessary to elucidate the potentiating mechanism of PRRSV against PCV2d.

In the dual infection model, the more virulent PCV2d strain replicated a higher level of viral blood load and induced more severe lymphoid lesions compared to the less-virulent PCV2a and PCV2b strains. Levels of PCV2 viremia was correlated to severity of lymphoid lesions. The evaluation of lymphoid lesions is critical in the determination of PCV2 strain virulence as prominent lymphoid lesions are one of the criteria of PCVAD. Unlike the PCV2 singularly infected pigs in the present study, those that were dually infected with PCV2 and PRRSV met the criteria for the typical of PCVAD. Therefore, PCV2 is the essential infectious agent of PCVAD but is not sufficient on its own to trigger the full clinical manifestations of PCVAD. Among potential triggers, PRRSV is considered a major risk factor in the induction of PCVAD in PCV2-infected pigs [16,17,27,28]. Pigs co-infected with PCV2d and PRRSV elicited more severe lymphoid lesions compared to pigs co-infected with either PCV2a and PRRSV, or PCV2b and PRRSV. The high virulence of pigs with PCV2d/PRRSV is clinically significant because PCV2d is the most predominant genotype currently circulating throughout Asia and North America. Nevertheless, no significant differences in ADWG were observed in the PCV2a/PRRSV, PCV2b/PRRSV, and PCV2d/PRRSV groups. Lack of the impact of the PCV2 genotypes in co-infection on ADWG may be due to short period of observation.

This experiment is to compare the virulence among the three PCV2 genotypes. Therefore, PRRSV used merely as co-factor to induce full manifestation of PCVAD. PCV2a and PCV2b did not potentiate the levels of PRRSV viral load in the blood or lung lesions in pigs dually infected with either PCV2a and PRRSV-2, or PCV2b and PRRSV-2 [17]. In addition, no significant differences in the levels of PRRSV-2 viremia were found in pigs dually infected with PCV2a/PRRSV-2, PCV2b/PRRSV-2, and PCV2d/PRRSV. ADWG was not also influenced by the PRRSV in co-infection model [19]. Since, the effect of PCV2 on PRRSV was not determined in this experiment, consequently, it was not included the pigs singularly infected with PRRSV as the Seoul National University Institutional Animal Care and Use Committee recommendation, in order to avoid unnecessary usage of pigs. Additional studies are necessary to determine the effect of PCV2 on PRRSV-2 including PRRSV-2-infected group.

Caution should be used in interpreting the results because only one strain for each of the three PCV2 genotypes was used in this experiment. Pigs co-infected with PCV2d and PRRSV-2 had higher levels of PCV2 loads in the blood and more severe lymphoid lesions compared to pigs co-infected with other PCV2 genotypes and PRRSV-2. Moreover, PRRSV-2 induced more severe PCVAD in co-infected pigs compared to PRRSV-1 [19]. Therefore, it is recommended swine practitioners and producers that the regular surveillance for the genotype of PCV2 and PRRSV is able to detect any potential risk for the introduction of new genotypes of viruses into pig herds.

## 4. Materials and Methods

### 4.1. Animals

Forty-two clinically healthy, colostrum-fed conventional pigs from sows that had not been previously vaccinated against PCV2 were purchased at 28 days of age from a commercial farm that was free of PRRSV. The farm was also *Mycoplasma hyopneumoniae* free based on serological testing, and long term clinical and slaughter history. The farm was seropositive for PCV2 but did not show PCVAD. Five sows used in the experiment were seronegative for PRRSV (HerdChek PRRS X3 Ab test, IDEXX Laboratories Inc., Westbrook, ME, USA), PCV2 (INgezim CIRCO IgG, Ingenasa, Madrid, Spain), and *M. hyopneumoniae* (*M*. *hyo*. Ab test, IDEXX Laboratories Inc). Five sows were confirmed negative for PCV2 (a, b, and d) and PRRSV in the blood, and *M. hyopneumoniae* in the larynx by real-time polymerase chain reaction (PCR). Sows were also negative for porcine parvovirus (PPV) 1 and 7 in blood by real-time PCR testing [29].

The pigs selected in the experiment were seronegative for PRRSV, PCV2, and *M. hyopneumoniae* using the same commercial ELISA. The same pigs were also confirmed negative for PCV2 (a, b, and d), PRRSV, PPV1 and PPV7 in the blood, and *M. hyopneumoniae* in the larynx by real-time PCR.

### 4.2. Experimental Design

For the study, pigs were allocated into 7 groups (6 pigs per group) using the random number generator function from Excel (Microsoft Corporation, Redmond, WA, USA) (Table 1). Pigs were randomly assigned into seven separate rooms based on group. At 0 dpi (42 days of age), pigs in the PCV2a, PCV2b, and PCV2d groups were inoculated intranasally with 3 mL of either PCV2a (SNUVR100032 strain, 5th passage in PCV-free PK15 cell lines, PCV-free PK15 cell line was kindly provided by WOOGENE B&G Ltd., Seoul, Korea), PCV2b (SNUVR202155 strain, 5th passage in PCV-free PK15 cell lines), or PCV2d (SNUVR202003 strain, 5th passage in PCV-free PK15 cell lines) inoculum containing 1.2 × 10^5^ 50% tissue culture infective dose (TCID_50_/mL), to their respective groups. Pigs in the PCV2a/PRRSV-2, PCV2b/PRRSV-2, and PCV2d/PRRSV-2 groups were each inoculated intranasally with a mixture of equal volume of PCV2 (PCV2a, PCV2b, and PCV2d) and PRRSV-2. Each 3mL PCV2 administered challenge contained 1.2 × 10^5^ TCID_50_/mL regardless of the PCV2 genotype as well as 3 mL of PRRSV-2 (SNUVR090851 strain) inoculum (1.2 × 10^5^ TCID_50_/mL). Infection of pigs with PRRSV-2 (SNUVR090851 strain) induced severe interstitial pneumonia in the lung [30]. Pigs in the negative control group were inoculated intranasally with 6 mL (3 mL/nostril) of uninfected cell culture supernatant.

Blood samples were collected from each pig by jugular venipuncture at 0, 7, 14, and 21 dpi. Pigs were sedated by an intravenous injection of sodium pentobarbital and then euthanized by electrocution at 21 dpi as previously described [31]. Tissues were collected from each pig at necropsy. All experimental protocols were approved prior to initiation of the study by the Seoul National University Institutional Animal Care and Use Committee (SNU-210120-7).

### 4.3. Clinical Observation

Pigs were monitored daily for clinical signs and scored weekly using a score ranking system which ranged from 0 to 6 (0 = normal; 1 = mild dyspnea or tachypnea or both when stress; 2 = mild dyspnea or tachypnea or both when at rest; 3 = moderate dyspnea or tachypnea or both when stress; 4 = moderate dyspnea or tachypnea or both when at rest; 5 = severe dyspnea or tachypnea or both when stress; 6 = severe dyspnea or tachypnea or both when at rest) [32]. All observers involved in these processes were blinded to type of challenge virus. Rectal temperatures were measured and recorded daily for 14 days after inoculation of PCV2 and PRRSV at the same time by same personnel.

### 4.4. Growth Performance

The live weight of each pig was measured at 42 (0 dpi) and 63 (21 dpi) days of age. The average daily weight gain (ADWG; gram/pig/day) was analyzed over the time period between 42 and 63 days of age. ADWG during the different production stages was calculated as the difference between the starting and final weight divided by the duration of the stage.

### 4.5. Quantification of PCV2 DNA in the Blood

A commercial kit (QIAamp DNA mini kit, QIAGEN, Valencia, CA, USA) was used to extract DNA from serum samples for PCV2. Genomic DNA copy numbers for PCV2a, PCV2b, and PCV2d were quantified by real-time PCR [33,34].

### 4.6. Quantification of PRRSV cDNA in the Blood

A commercial kit (QIAamp viral RNA mini kit, QIAGEN) was used to extract RNA from serum samples for PRRSV-2, as previously described [29]. Real-time PCR were performed to quantify PRRSV-2 genomic cDNA copy [35].

### 4.7. Serology

Serum samples were tested for antibodies against PCV2 (INgezim CIRCO IgG, Ingenasa, Madrid, Spain) and PRRSV (HerdChek PRRS X3 Ab test, IDEXX Laboratories Inc.). Samples were considered positive for PCV2 antibodies if the optical density (OD) was greater than 0.3 and for PRRSV antibodies if the OD was greater than 0.4 according to the manufacturer’s instructions.

### 4.8. Histopathology

For the morphometric analysis of histopathological changes in superficial inguinal lymph nodes, three sections of that lymph node were examined “blindly” [36]. Lymph nodes were evaluated for presence of lymphoid depletion and inflammation, and given a score ranging from 0 to 5 (0 = normal; 1 = mild lymphoid depletion; 2 = mild to moderate lymphoid depletion and histiocytic replacement; 3 = moderate diffuse lymphoid depletion and histiocytic replacement; 4 = moderate to severe lymphoid depletion and histiocytic replacement; 5 = severe lymphoid depletion and histiocytic replacement).

### 4.9. Immunohistochemistry

Immunohistochemistry (IHC) and morphometric analysis of IHC was carried out as previously described [37]. Positive signal was quantified using the NIH Image J 1.45s Program (http://imagej.nih.gov/ij/download.html, accessed on 12 July 2021). For each slide of lymph node tissue, 10 fields were randomly selected, and the number of positive cells per unit area (0.25 mm^2^) was counted. The mean values were also calculated [37].

### 4.10. Statistical Analysis

Prior to statistical analysis, real-time PCR data were log-transformed to reduce variance and positive skewness. Data were tested for normal distribution using the Shapiro–Wilk test. A one-way analysis of variance (ANOVA) was used to examine whether significant statistical differences existed among the seven groups for each time point. When a one-way ANOVA test result showed a statistical significance, a post hoc test was conducted for a pairwise comparison with Tukey’s adjustment. If the normality assumption was not met, the Kruskal–Wallis test was performed. When the result from the Kruskal–Wallis test showed statistical significance, a Mann–Whitney test with Tukey’s adjustment was performed to compare the differences among the groups. Spearman’s (non-normally distributed variables) correlations were applied to determine the relationship between the levels of PCV2 viremia and severity of lymphoid lesions. A value of *p* < 0.05 was considered to be significant.

## Figures and Tables

**Table 1:** Body weight and average daily weight gain (ADWG) data (mean ± standard deviation) from pigs dually inoculated with porcine circovirus type 2 (PCV2) and porcine reproductive and respiratory syndrome virus-2 (PRRSV-2) and pigs singularly inoculated with PCV2 at 42 days of age (0 days post-inoculation, dpi) and 63 days of age (21 dpi).

	Body Weight (kg)		ADWG
Groups	42 Days Old (0 dpi)	63 Days Old (21 dpi)	Between 42 and 63 Days Old
PCV2a/PRRSV-2	8.47 ± 0.26	13.17 ± 0.71 ^a^	228.81 ± 34.45 ^a^
PCV2b/PRRSV-2	8.55 ± 0.28	13.53 ± 0.87 ^a^	237.30 ± 41.45 ^a^
PCV2d/PRRSV-2	8.32 ± 0.46	12.77 ± 0.68 ^a^	211.90 ± 27.56 ^a^
PCV2a	8.35 ± 0.10	16.03 ± 0.53 ^b^	365.87 ± 28.48 ^b^
PCV2b	8.42 ± 0.24	16.07 ± 0.73 ^b^	364.29 ± 33.37 ^b^
PCV2d	8.50 ± 0.23	16.22 ± 0.63 ^b^	367.46 ± 26.33 ^b^
Negative control	8.42 ± 0.26	16.17 ± 0.46 ^b^	369.05 ± 17.24 ^b^

Different superscripts (a and b) indicate significant (*p* < 0.05) difference among 7 groups.

**Table 2:** Pathology data (mean ± standard deviation) from pigs dually inoculated with porcine circovirus type 2 (PCV2) and porcine reproductive and respiratory syndrome virus-2 (PRRSV-2) and pigs singularly inoculated with PCV2 at 21 days post-inoculation.

Groups	Microscopic Lung Lesion Scores	Microscopic Lymphoid Lesion Scores	PCV2-Antigen Positive Cells within Lymphoid Lesion
PCV2a/PRRSV-2	2.73 ± 0.52 ^a^	3.07 ± 0.39 ^a^	30.06 ± 4.57 ^a^
PCV2b/PRRSV-2	2.80 ± 0.47 ^a^	3.13 ± 0.27 ^a^	30.94 ± 4.50 ^a^
PCV2d/PRRSV-2	3.47 ± 0.43 ^b^	3.63 ± 0.23 ^b^	38.50 ± 4.37 ^b^
PCV2a	1.27 ± 0.24 ^c^	1.40 ± 0.18 ^c^	18.44 ± 3.27 ^c^
PCV2b	1.37 ± 0.20 ^c^	1.40 ± 0.22 ^c^	19.39 ± 4.68 ^c^
PCV2d	1.33 ± 0.30 ^c^	1.37 ± 0.23 ^c^	18.83 ± 4.49 ^c^
Negative control	0 ± 0	0 ± 0	0 ± 0

Different superscripts (a, b, and c) indicate significant (*p* < 0.05) difference among 7 groups.

## Data Availability

The data present in the study are available on request from the corresponding author.
